# A Practical Method for QTc Interval Measurement

**DOI:** 10.7759/cureus.12122

**Published:** 2020-12-17

**Authors:** Nestor R De Oliveira Neto, William Santos De Oliveira, Guilherme D Campos Pinto, Eric Santos R De Oliveira, Maria das Neves D Da Silveira Barros

**Affiliations:** 1 Cardiology, Hospital Universitário Onofre Lopes, Natal, BRA; 2 Cardiology, University of Pernambuco (UPE), Recife, BRA

**Keywords:** qt interval, qtc, qt correction, hodges, bazett, qt calculation

## Abstract

Objective

The various formulae used for QT correction by heart rate (HR) require the execution of operations with the aid of calculators or applications. This study aimed to evaluate the performance of a simple rule for QTc estimation, comparing the measurements obtained with those provided by the commonly used equations of Bazett, Fridericia, Framingham, and Hodges.

Methods

We used the database of a previous observational study, which analyzed patients prospectively with acute pulmonary edema admitted in an emergency service. One hundred four patients were included for QTc assessment, of whom 86 patients underwent two ECG: one ECG <24h and other >24h after admission. Thus, a total of 190 ECGs were analyzed by two observers that manually measured QT and HR. QTc was obtained using the known formulae and the proposed equations: QTc = QT+2 (FC-60) for HR ≤ 90 bpm and QTc=QT+2(FC-60)-10 for HR>90 bpm.

Results

Bland-Altman plots show good agreement between the simple rule and Hodges equation, with a mean difference of -3,4, SD of 4.96 and 95% limits of agreement from -9,9 to 3.2. There was not a good agreement between the simple method and the other formulae.

Conclusion

The proposed method has good agreement with the measures of QTc by the equation of Hodges in the HR range of 40 to 130bpm in acutely ill patients. Our method may be a plausible option for quick QT correction in these subjects.

## Introduction

The QT interval, measured from the beginning of the QRS to the end of the T wave, corresponds to the ventricular electrical systole. QT interval measurement is a parameter of prognostic importance in many medical conditions, and a lengthening in QT is associated with higher mortality in extensive observational studies [1,2]. Likewise, medications used in everyday practice can lead to an abnormally prolonged QT interval and torsades de pointes-type arrhythmias and sudden cardiac death [3,4]. However, as the QT is inversely proportional to the heart rate (HR), the corrected QT (QTc) interval calculation is necessary to adjust QT values for a standard HR. Several formulae have been proposed for calculating the QTc interval, such as Bazett, Fridericia, Framingham, and Hodges equations.

The competence for ECG interpretation is known to be suboptimal among interns and resident physicians, with low levels of correct answers and high variability with the primary electrocardiographic diagnoses [5,6]. QTc calculation should be part of ECG interpretation as it requires the execution of arithmetic operations with the aid of calculators or applications [7,8].

This study aimed to test the performance of a simple rule for determining the QTc interval, comparing the measurements obtained by this method with those provided by the commonly used equations and also to evaluate the agreement between the differences of the QTc (ΔQTc) between successive tracings of the same patient (ECG 1 and 2) as calculated using the simple rule and the reference equations.

## Materials and methods

Study Population

This study used the database generated by a previous observational diagnostic study [9], which analyzed prospectively 150 patients with acute pulmonary edema, between 40 and 80 years of age, consecutively admitted to a public emergency service specialized in cardiology. The ethics committee approved this observational study of our hospital (PROCAPE/UPE), and the consent form was waived because it was a retrospective study.

All patients underwent serial 12-lead ECG, Doppler echocardiography and cardiac catheterization.

We excluded patients with either wide QRS or irregular rhythm, as the presence of wide QRS (≥120 ms) causes QT prolongation and requires additional corrections to determine QTc; similarly, in the presence of an irregular rhythm, the measured QT varies from beat to beat in proportion to the previous RR interval and the average RR, challenging the accurate calculation of QTc [10,11].

Electrocardiogram and QT Measurements

ECGs were performed on the first and second days of hospitalization (ECG 1 e 2) and were analyzed by two observers with experience in ECG interpretation (NRON and MNDSB).

ECG was recorded on a standardized paper with at 10 mm/mV and paper speed of 25 mm/s, with patients at rest and on supine position, using a 3-channel machine (Dixtal Cardio-page EP-3, Dixtal Biomedica, São Paulo, Brazil).

The QT interval measurement was made visually using the lead with greater QT values, usually V2, V3, II, or V5, and using the RR interval that preceded the measured QT interval. When the T wave ending was unclear or overlapped with the U wave, a tangent line following T wave slope was drawn to the baseline. To set the values of QT and RR, we used the average QT and RR of 3 beats at a stable sinus rhythm.

According to the analysis of QTc value curves obtained by Luo et al. [8] using the equations of Bazett, Hodges, Fridericia and Framingham in the heart rate (HR) range of 40 to 140bpm for correction of QT values of 350 and 500, we observed that the following equation provides QTc values very close to those obtained by Hodges: QTc = QT + 2 (HR - 60) for FC ≤ 90bpm, and QTc = QT + 2 (HR - 60) - 10 for HR > 90bpm.

Based on this last equation, the following method was used to estimate the QTc: we selected a QRS complex, on the standard ECG, at the usual speed of 25mm/s, and we measured the QT interval in small squares. The heart rate (HR) is determined (HR =1500/RR), where RR is measured in the previous cycle. To find the QTc value, the QT is multiplied by 40 ms (QT measured in ms), and then we add twice the difference between the HR and 60bpm (2 x (HR-60)). In the case of HR > 90bpm, the value is reduced by 10ms.

All QTc values were measured in milliseconds (ms).

Statistical Analysis

Data analysis was performed with MedCalc for Windows, version 15.0 (MedCalc Software, Ostend, Belgium) and Excel version 14.1.0 for macOS (Microsoft Corp., Redmond, WA, USA), considering a type I error of 0.05 and a type II error of 0.20, limits of agreement of 95%. All continuous variables are given as mean and standard deviation, and proportions as percentages.

QTc/HR graphs were constructed for each QTc formula, with the QTc on the y-axis (in ms) and the HR on the x-axis (in bpm), obtaining the linear regression slope in each case and performing comparative analysis between them by ANOVA. The equation with a slope value close to zero has better performance, indicating less influence of HR on QTc.

Bland-Altman plots with a calculation of bias and limits of agreement were performed between the QT values calculated by the tested rule and the formulas of Bazett, Fridericia, Framingham and Hodges.

Likewise, an agreement between the differences in QTc (ΔQTc) in successive tracings of the same patient (ECG 1 and 2) was also made by the Bland-Altman plot, comparing ΔQTc values obtained between the different methods, using the same formula before and after.

For evaluation of interobserver reproducibility, Bland-Altman analysis and concordance tests were performed in a random sample of 25 ECGs.

## Results

Of the initial sample of 150 patients admitted with acute pulmonary edema in the emergency unit, in 141 patients, the ECGs tracings were adequate to measure QT and HR. Of these, 37 patients were excluded for presenting left bundle block (26), atrial fibrillation or atrial tachycardia (7), right bundle block (4) and atrial fibrillation and left bundle block (1). Of the 104 remaining patients, 86 underwent an initial ECG (<24 h) and another after 24 h. Thus, a total of 190 ECGs were available for QTc assessments.

In the sample, the HR ranged from 44 to 150bpm, with an average of 92±23 beats/min. This HR reflects the profile of the evaluated population (i.e., patients with acute pulmonary edema seen in an emergency unit) and allowed for evaluating the QTc measurements obtained by the simple method and other equations in a wide range of HR. Clinical and demographic characteristics of the studied patients are shown in Table 1.

**Table 1:** Clinical and demographic characteristics of the studied patients.

Characteristic	Total (150)
Age (years)	65.5±10.5
Male sex	36%
History of diabetes	49%
History of hypertension	79%
History of smoking	55%
Presence of coronary artery disease	60%
Heart rate (bpm)	92±23
Ejection fraction	47.8±15.1
Ejection fraction <40%	34%
Positive troponin ng/mL	93%
Troponin ng/mL (1st measurement)	0.43±1.12
Length of hospital stay (days)	8.3±7.2
Hospital death	14%

**Figure 1:**
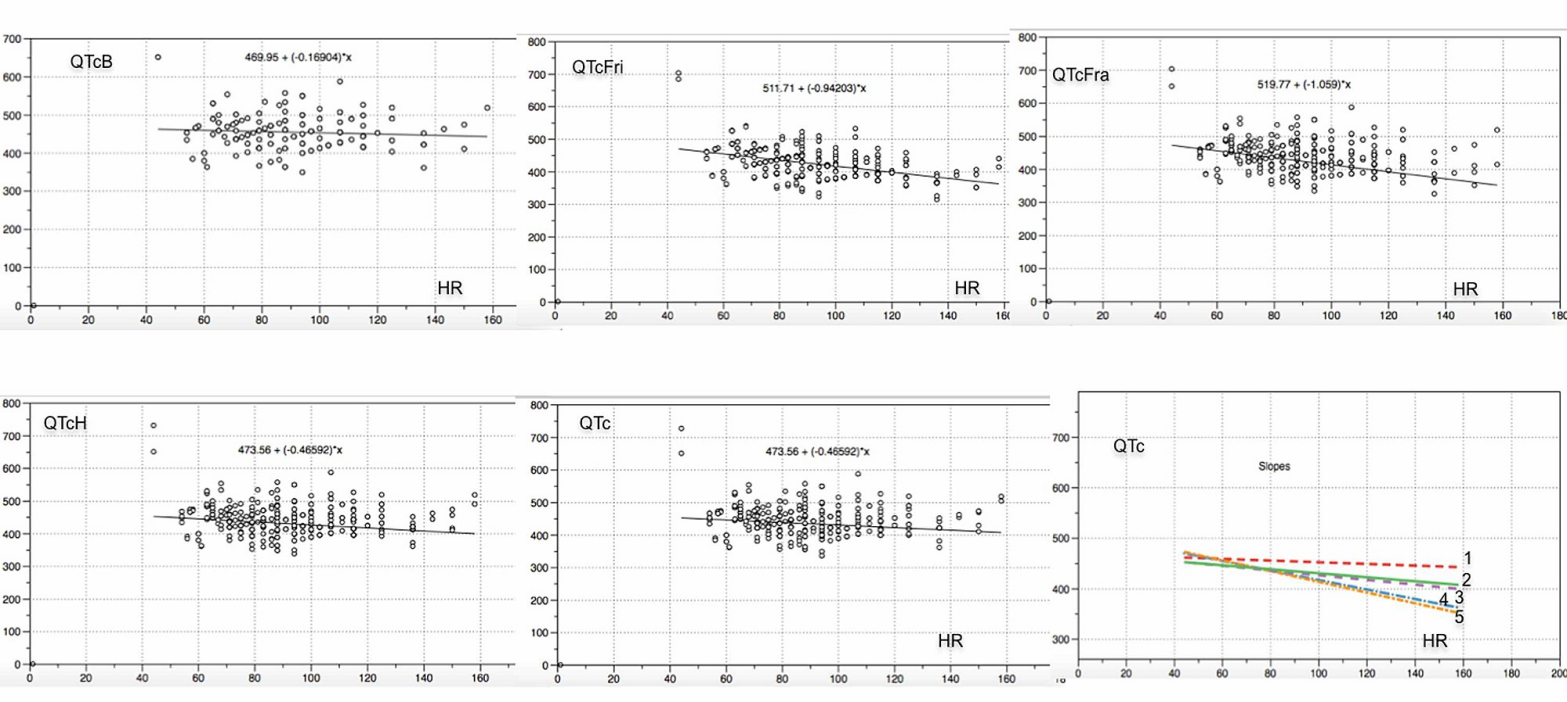
QTc/HR Plot QTc/HR plot and linear regression slope in the studied patients, using the values ​​of QTc corrected by heart rate (HR) as calculated by Bazett (QTcB), Fridericia (QTcFri), Framingham (QTcFram), Hodges (QTcH) and simple method (QTcS). Fridericia and Framingham formulas have higher slopes and correlation coefficients. The slopes of the curves of Bazett (1), simple method (2) and Hodges (3) were less pronounced.

We observed lower correlation coefficients (R2) and slope of the QTc/HR linear regressions using Bazett, Hodges and the simplified method, compared to the dates derived from Fridericia and Framingham in our sample. QTc/RR plot and linear regression slope of the different correction formulae in the studied patients are shown in Figure 1.

**Figure 2:**
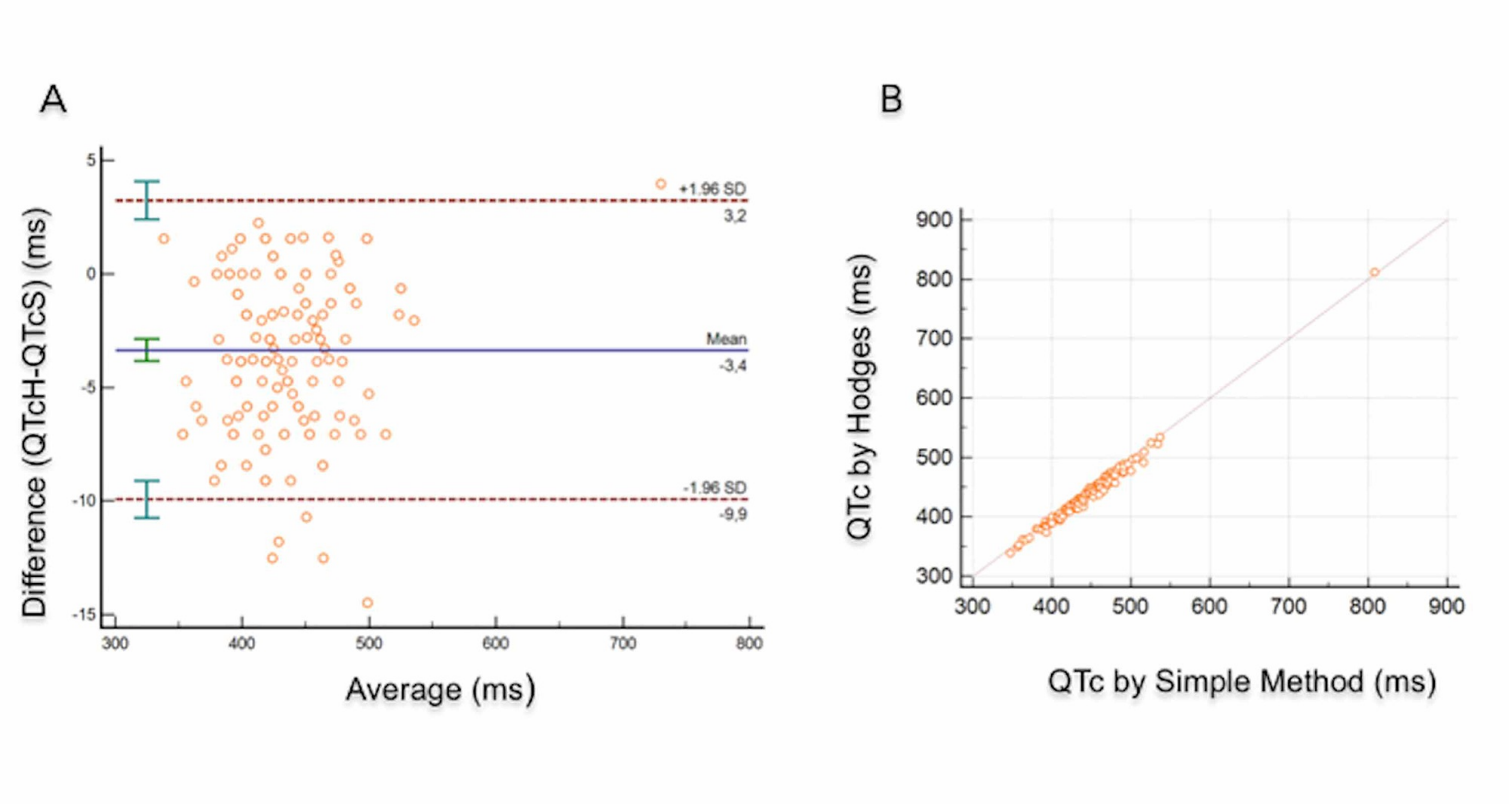
Bland-Altman Plot Between Hodges QTc and QTc Values by the Proposed Simple Method There is a good agreement between QTc measurements by Hodges and the proposed simple method (A) as well as a high concordance correlation coefficient between results given by both equations (B).

Bland-Altman plots with the calculation of bias and limits of agreement between a simple method and the Hodges formula show good agreement between the two measurements (Figure 2), with a mean difference (bias) of -3,4, SD of 4.96 and 95% limits of agreement from -9,9 to 3.2. In only three cases, the difference between the QTc measured by Hodges and the proposed method was greater than 10 ms, all with HR of 136 bpm or greater; the maximum QTc difference was of 14,47 ms with an HR of 158 bpm. Bland-Altman analyzes between QTc correction formulas are shown in Table 2. In general, there was not a good agreement between the simple method and the other formulas, nor between these formulas among themselves, except between Framingham and Fridericia.

An acceptable correlation was found for intra-observer measurements of QTc values ​​by the simplified method, according to the Bland-Altman plot, with a mean ± SD bias of -1.6 ± 17.6 and 95% LOA from -32.9 to 36.0.

Adopting a normal QTc range of up to 0.45 in men and 0.46 in women, there is a higher percentage of patients with prolonged QTc in our sample when the correction is performed by Bazett. Accordingly, the QTc was prolonged in 42% of cases by Bazett, which was significantly higher than when the correction was made using the other formulas: Fridericia (23%, p = 0.0011); Framingham (20%, p = 0.0001); Hodges: (22%, p = 0.0005) and by the simple rule: (23%, p = 0.0016).

Serial QTc analysis is important to monitor the effect of drugs and the risk of sudden death [3,4]. In this study, 86 patients had ECGs separated by 24 h or more, which allowed assessing the degree of increase or decrease in QTc between the two successive tracings. Thus, the determination of the QTc delta was carried out using the different formulas. Again, there is good agreement between the delta QTc values ​​calculated by the new method compared to those corrected by Hodges' formula (mean ± SD bias: 0.87 ± 4.96, 95% LOA range: -8.85 to 10.58.

On the other hand, in general, there was a considerable discrepancy between the delta QTc values measured by the formulas. There was low agreement between the measures taken by Bazett and the other formulas. For instance, the agreement between Bazett and Hodges measurements showed a mean ± SD bias of 0.38 ± 18.67 and 95% LOA from -36.1 to 36.89 (Table 2).

**Table 2:** Bland Altman's comparative analysis of the QTc between some formulae. SD: standard deviation, LOA: limits of agreement.

Bland-Altman Comparison	Bias (ms)	SD (ms)	95% LOA
Bazett x Fridericia	28.13	17.09	-5.36 to 61.63
Bazett x Hodges	23.08	15.28	-6.86 to 53.03
Bazett x Simple Method	19.73	15.16	-9.98 to 49.44
Hodges x Framingham	7.61	14.89	-21.57 to 36.79
Hodges x Simple Method	-3.35	3.36	-9.94 to 3.23
Framingham x Fridericia	-2.56	5.81	-13.91 to 8.83
Fridericia x Simple Method	8.40	15.43	-38.64 to 21.83

## Discussion

Accurate QTc calculation involves several challenges, such as determining the QT interval ending on surface ECG and choosing the appropriate formula for performing heart rate correction with the use of calculators [10]. Thus, we developed a method for rapid assessment of QTc with good agreement with Hodges' linear formula, presenting few outliers in the heart rate of 40 to 130 bpm (by Bland-Altman plot) in a population of patients admitted with acute pulmonary edema.

**Figure 3:**
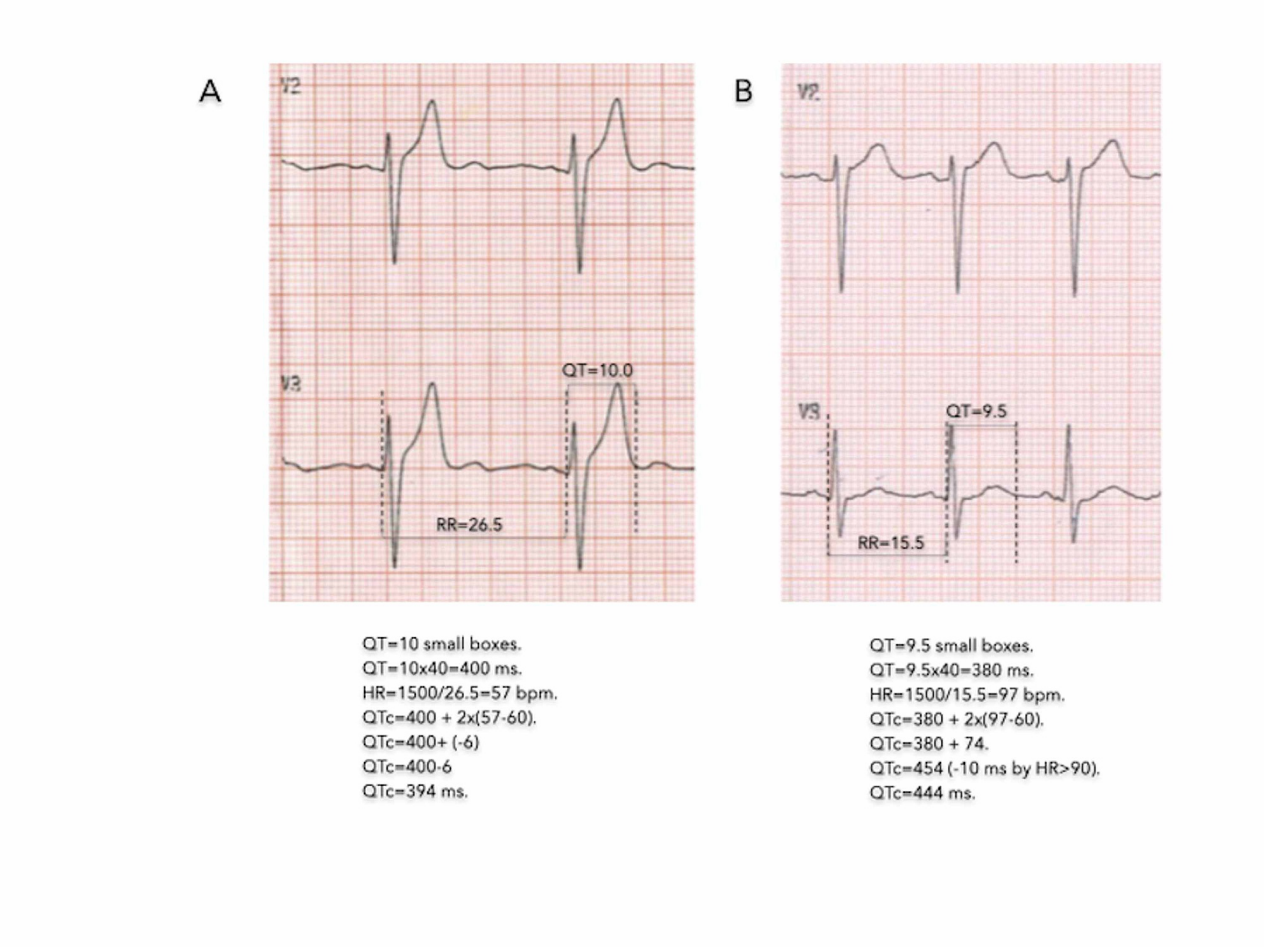
Examples of how QT was measured and corrected by HR using the proposed method. Examples of how QT was measured and corrected by heart rate (HR) using the proposed method. The QT in millimetres is multiplied by 40ms to find the QT in milliseconds (ms). Then the HR is determined (HR = 1500/RR). The QTc is obtained by adding the measured QT to twice the difference: HR-60. In the case of HR> 90 bpm, the value is reduced by 10 ms. A) QTc= 394ms. Note that if HR<60bpm, the subtraction (HR-60) gives a negative number, so the QTc is less than the measured QT. The value of QTc by Hodges is equal to 395ms. B) QTc=444ms. As the HR> 60bpm, the difference (HR-60) is positive, so the QTc is greater than the measured QT. The QTc by Hodge is 445ms.

In general, there was not a good agreement between the simple method and the other formulas, nor between these formulas among themselves, except between Framingham and Fridericia. In figure 3, we have an example of how we perform QT correction by HR using the proposed method.

Examples of how QT was measured and corrected by HR using the proposed method. The QT in millimetres is multiplied by 40ms to find the QT in milliseconds (ms). Then the heart rate (HR) is determined (HR = 1500/RR). The QTc is obtained by adding the measured QT to twice the difference: HR-60. In the case of HR> 90 bpm, the value is reduced by 10 ms. A) QTc= 394 ms. Note that if HR<60 bpm, the subtraction (HR-60) gives a negative number, so the QTc is less than the measured QT. The value of QTc by Hodges is equal to 395 ms. B) QTc=444 ms. As the HR> 60 bpm, the difference (FC-60) is positive, so the QTc is greater than the measured QT. The QTc by Hodge is 445 ms.

It is still unclear, which is the best equation for correcting QT [8,11-14]. The ideal QT correction method should present slope value and R2 coefficient close to zero, which means that the HR would be of little influence over the corrected value. A comparison of four formulae in 10.303 normal ECG from an American database [8] of healthy individuals showed QT correction per Bazett to present a significantly wider distribution than per Fridericia, Framingham, and Hodges formulae. Accordingly, despite being the most used formula for QT correction, Bazett presented the most significant dependence upon heart rate, leading to overcorrection of QT interval at high heart rates and under-correction at low heart rates. These concerns have led a 2009 AHA/ACCF/HRS Scientific Statement [7] to recommend the use of linear regression formulae (e.g. Framingham, Hodges) rather than Bazett's formula for QT-rate correction. In our study, however, Bazett's QTc values presented lower linear regression slope and R2 coefficient than other equations. This likely reflects the particularity of our studied population, most of whom were elders with coronary artery disease. Accordingly, the QT/RR linear regression is highly variable among healthy individuals [15], patients with previous myocardial infarction [16], and also between individuals with either cardioembolic stroke or atherosclerotic stroke [16]. Hence, the QT/RR relation may be heavily influenced by the patient characteristics of each database [15-17].

Additionally, there were about twice as many patients with prolonged QTc values in our sample when QT correction was performed by Bazett instead of other equations. Bazett's formula is known to generate higher QTc values compared to other formulae when the HR is above 60 bpm [8], in order that the high HR in our evaluated database likely contributed to this finding. Accordingly, in the sample analyzed by Luo et al. [8], 10% of people with an apparently normal ECG had a QTc > 460 ms by Bazett, whereas this occurred in only 2% of individuals when other formulae adjusted the QTc.

Modern digital ECG machines automatically measure the QT and HR intervals, as well as calculate the QTc itself. However, in resource-poor settings, ECG machines without algorithms for performing automatic measurements are widely used. Also, these algorithms can be inaccurate, and thus the QTc needs to be calculated and confirmed by the physician. The "Half of RR" rule has been proposed as an initial screening method for assessing the QTc [18], but it has been shown to perform poorly compared to other methods of QT correction [19]. Our simple method seems to estimate the QTc based on the surface ECG analysis quickly. It presents good agreement with the QT correction as calculated by the well-known Hodges formula.

Limitations

The main limitation of our study is the absence of a control group of healthy individuals for determining the performance of our proposed method in comparison with the group of acutely ill patients. Additionally, as most published studies of QT correction formulae have used healthy individuals in their databases, the presence of such a patient group would have enabled a more reliable comparison with previous data. Other limitations include the retrospective nature of our study and the fact that it used a database of individuals with acute pulmonary edema in a single institution. Our findings may not apply to patients with other conditions.

## Conclusions

We propose a simple rule for practical correction of QT by heart rate. Our method has good agreement with the measures of QTc by the linear equation of Hodges in the HR range of 40 to 130 bpm and may aid as an initial tool in emergency units or resource-poor settings. As we used a database of patients with acute pulmonary edema, further data would ideally assess our method in healthy individuals and patients with other medical conditions.
